# The Protective Effects of *Reineckia carnea* Ether Fraction against Alzheimer’s Disease Pathology: An Exploration in *Caenorhabditis elegans* Models

**DOI:** 10.3390/ijms242216536

**Published:** 2023-11-20

**Authors:** Hai-Jun Fu, Xing-Yue Zhou, Ya-Ping Li, Xue Chen, Yan-Ni He, Da-Lian Qin, Lu Yu, Chong-Lin Yu, Jian-Ming Wu, An-Guo Wu, Xiao-Gang Zhou

**Affiliations:** 1 Sichuan Key Medical Laboratory of New Drug Discovery and Drugability Evaluation, School of Pharmacy, Southwest Medical University, Luzhou 646000, China; fffh0505@163.com (H.-J.F.); 13980258029@163.com (X.-Y.Z.); cx790465521@163.com (X.C.); yannihejnu@163.com (Y.-N.H.); dalianqin@swmu.edu.cn (D.-L.Q.); yulu863@swmu.edu.cn (L.Y.); 8056ycl@swmu.edu.cn (C.-L.Y.); jianmingwu@swmu.edu.cn (J.-M.W.); 2 Central Nervous System Drug Key Laboratory of Sichuan Province, Luzhou 646000, China

**Keywords:** Alzheimer’s disease, *Reineckia carnea* ether fraction, autophagy, Aβ, Tau

## Abstract

Alzheimer’s disease (AD) presents a significant challenge to global healthcare systems, with current treatments offering only modest relief and often bringing unwanted side effects, necessitating the exploration of more effective and safer drugs. In this study, we employed the *Caenorhabditis elegans* (*C. elegans*) model, specifically the AD-like CL4176 strain expressing the human Aβ_(1–42)_ protein, to investigate the potential of *Reineckia carnea* extract and its fractions. Our results showed that the *Reineckia carnea* ether fraction (REF) notably diminished the paralysis rates of CL4176 worms. Additionally, REF also attenuated the neurotoxicity effects prompted by Tau proteins in the BR5270 worms. Moreover, REF was observed to counteract the accumulation of Aβ and pTau proteins and their induced oxidative stress in *C. elegan*s AD-like models. Mechanistic studies revealed that REF’s benefits were associated with the induction of autophagy in worms; however, these protective effects were nullified when autophagy-related genes were suppressed using RNAi bacteria. Together, these findings highlight *Reineckia carnea* ether fraction as a promising candidate for AD treatment, warranting further investigation into its autophagy-inducing components and their molecular mechanisms.

## 1. Introduction

As global populations age and life expectancy increases, the implications of aging and its related diseases have become a focal point of medical research. Alzheimer’s disease (AD), characterized as the most common type of dementia, is an age-associated neurodegenerative disorder [1]. It affects approximately 5% of individuals over 60 years of age, and its prevalence sharply escalates with advancing age. At present, an alarming 50 million people globally suffer from dementia, with AD accounting for 35 million of these cases [2]. This epidemic imposes a substantial economic and medical burden on global healthcare infrastructures. Despite extensive research identifying β-amyloid (Aβ) deposition in senile plaques and hyperphosphorylated Tau (pTau) in intracellular neurofibrillary tangles as key pathological markers of AD, approved treatments remain limited [3]. Moreover, the drugs currently available often come with significant side effects and offer only modest symptomatic relief for mild-to-moderate AD patients [4]. Thus, the quest for more efficacious and safer therapeutic options for AD remains crucial.

Emerging research suggests that natural products, particularly those found in traditional Chinese medicines (TCMs), hold promise for treating aging-related disorders like AD [5]. For instance, the ethanol extracted from *Citri Reticulatae* semen has been shown to promote healthy aging and provide neuroprotective effects by inducing autophagy in *C. elegans* [6]. Similarly, water extracts from *Salvia miltiorrhiza* have also demonstrated potential neuroprotective properties against AD in *C. elegans* models, which has been attributed to their antioxidant activities [7]. These findings have catalyzed a growing interest in the exploration of natural products or their active compounds as prospective agents to delay the aging process and treat age-associated diseases. Among these, *Reineckia carnea* (Andr.) Kunth (referred to herein as *R. carnea*), an evergreen medicinal herb known as “Jixiangcao” in Chinese, has been employed for centuries to treat a range of conditions, including coughs, sore throats, traumatic injuries, and bronchial asthma [8]. Comprehensive phytochemical analyses have revealed that *R. carnea* contains an array of bioactive components such as saponins, flavonoids, volatile oils, and alkaloids [9]. These constituents have been studied for their varied pharmacological activities, including anti-inflammatory, antitussive, anticancer, anticomplement, and antioxidant properties [10,11,12]. For example, the steroidal saponin RCE-4, a major active component of *R. carnea*, has been found to effectively induce apoptosis in cervical cancer cells [13]. Furthermore, certain steroidal glycosides isolated from *R. carnea* have shown promising in vitro antitussive activity [14]. However, the potential role and underlying molecular mechanisms of *R. carnea* in mitigating AD remain largely unexplored.

The nematode *Caenorhabditis elegans* (*C. elegans*) has risen to prominence as a model organism in the study of aging-related diseases, especially neurodegenerative diseases (NDs) [15]. This preference stems from its several beneficial characteristics, which include a brief lifespan and reproductive cycle, compact size, transparent body, ease of upkeep and drug application, along with a well-mapped cell lineage and anatomy [16]. Of particular significance, *C. elegans* boasts a fully-functional and manageable nervous system composed of 302 neurons linked by approximately 5000 chemical synapses [17]. This makes it a valuable tool for observing neurotoxic responses. A prominent application involves the use of transgenic AD worms, specifically the CL4176 strain, which express the human Aβ_(1–42)_ protein in their body-wall muscle cells [18]. These worms are extensively utilized to preliminarily assess the impact of drugs on Aβ pathology, allowing efficient and precise methodologies to evaluate drug effects [19]. In this research, we utilized CL4176 worms to delve into the anti-AD potentials of *R. carnea* methanol extract (RE) and its fractions. Our findings revealed that the *R. carnea* ether fraction (REF) was particularly effective in reducing the paralysis rate, outperforming equal concentrations of RE and other fractions. Further analysis revealed that REF not only attenuated the toxic effects induced by Aβ and Tau but also reduced their protein levels in the *C. elegans* AD models. Concurrently, REF minimized the oxidative stress originating from the aggregation of Aβ and Tau in the worms. Intriguingly, we observed a notable activation of autophagy in worms treated with REF. However, when we employed RNA interference (RNAi) to suppress autophagy genes, the protective effects of REF against Aβ and Tau toxicity were neutralized. In summation, our findings shed light on the mechanisms through which REF from *R. carnea* provides protection against AD. These insights pave the way for further exploration and potential therapeutic applications of REF in AD treatment.

## 2. Results

### 2.1. Identification of Bioactive Constituents in R. carnea

**Figure 1:**
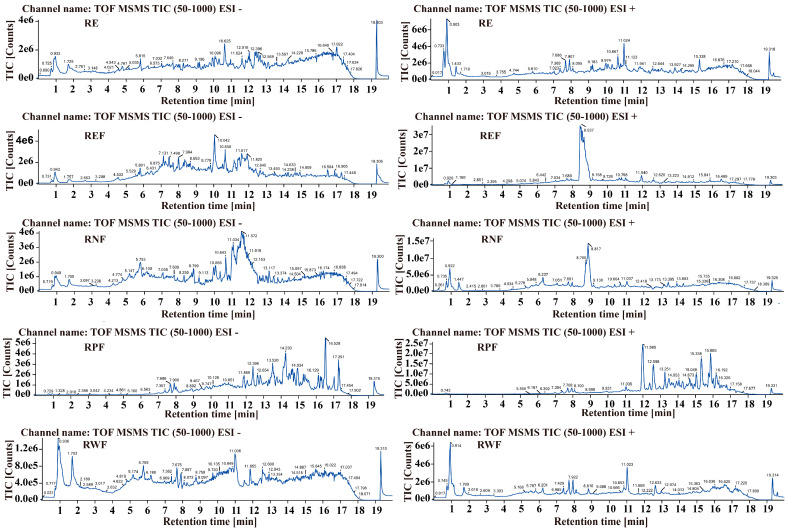
UPLC/Q-TOF-MS/M analysis of constituents in *R. carnea* extract and its fractions. (**Right**): Total ion chromatograms (TICs) representing RE, REF, RNF, RWF, and RPF in negative ion mode. (**Left**): TICs representing RE, REF, RNF, RWF, and RPF in positive ion mode.

The analysis of the methanol extract and various fractions of *R. carnea* was carried out using UPLC/Q-TOF-MS/MS. Representative chromatograms for this analysis are illustrated in Figure 1. A comprehensive list of the components identified in *R. carnea*—including their chemical names, molecular formulas, masses, and retention times—is detailed in Table 1. The primary constituents include a diverse range of organic acids, amino acids, polysaccharides, flavonoids, and lipids, such as L-Valine, L-Leucine, Thymidine, hexadecanamide, Palmitic acid, and Linolenic acid. These findings are in alignment with the components identified in previous studies [9,20].

### 2.2. REF Inhibits Aβ-Induced Paralysis and Tau-Induced Neurotoxicity in C. elegans

**Figure 2:**
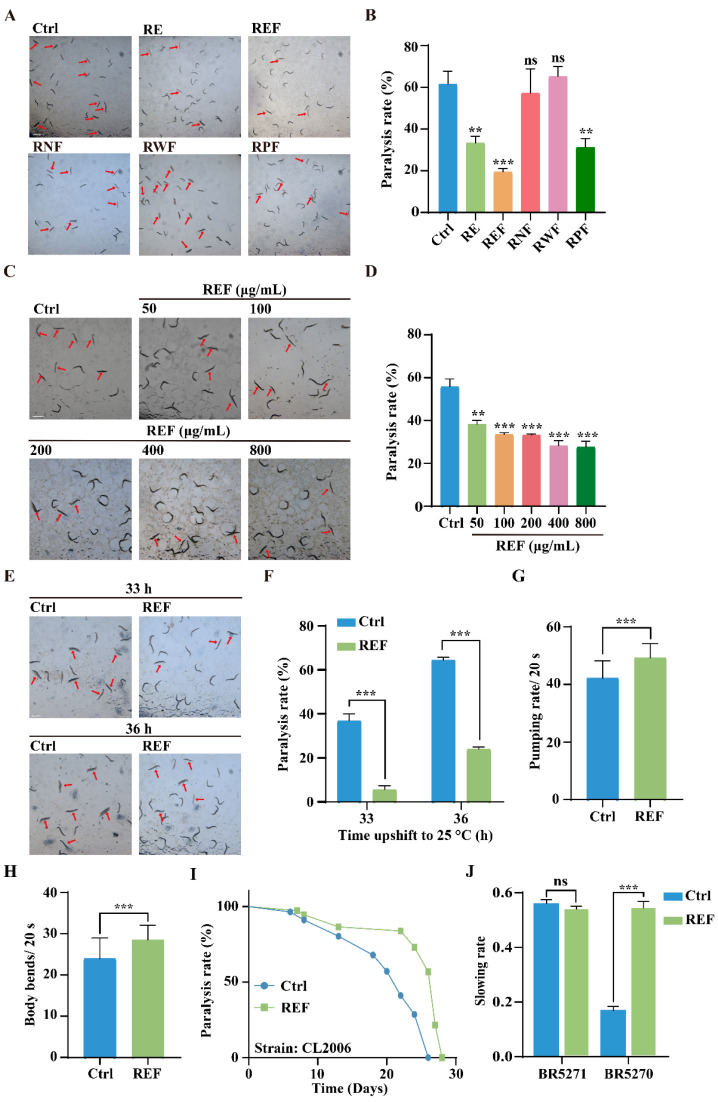
REF attenuates Aβ-induced paralysis and Tau-induced neurotoxicity in *C. elegans*. (**A**,**B**) CL4176 worms were treated with RE (100 μg/mL), REF (100 μg/mL), RNF (100 μg/mL), RWF (100 μg/mL), and RPF (100 μg/mL). Representative photographs indicate the paralysis of CL4176 worms after transfer to 25 °C for 30 h. Paralyzed worms are indicated by red arrows. Scale bar: 1 mm. Bar graph illustrates the paralysis rate of CL4176 worms. Data: mean ± SD (n > 60 nematodes per group), ns *p* > 0.05, ** *p* < 0.01, *** *p* < 0.001. (**C**,**D**) CL4176 worms were treated with varying REF concentrations. Representative photographs show the paralyzed worms, and the bar graph denotes the paralysis rate of worms. Scale bar: 1 mm. Data: mean ± SD (n > 60 nematodes per group), ** *p* < 0.01, *** *p* < 0.001. (**E**,**F**) CL4176 worms were treated with REF for 33 h and 36 h. Bar graph shows the paralysis rate of worms. Scale bar: 1 mm. Data: mean ± SD (n > 60 nematodes per group), *** *p* < 0.001. (**G**,**H**) Bar graphs display pumping rate and body bends of REF-treated CL4176 worms in 20 s. Data: mean ± SD (n = 27 nematodes per group), *** *p* < 0.001. (**I**) Line graph illustrates paralysis rate of REF-treated CL2006 worms. (**J**) Bar graph shows slowing rate of BR5271 and BR5270 worms. Data: mean ± SD (n = 15 nematodes per group), ns *p* > 0.05, *** *p* < 0.001.

We initially investigated the effect of *R. carnea* extract and its fractions on the inhibition of Aβ-induced toxicity using the transgenic CL4176 strain of *C. elegans*. This strain exhibits a temperature-sensitive expression of human Aβ_(1–42)_ protein in their body-wall muscle cells, governed by the muscle-specific *unc-54* promoter [18]. Such expression leads to the buildup of toxic aggregates, eventually manifesting as progressive paralysis. Our data, illustrated in Figure 2A,B, indicated that REF at a concentration of 100 μg/mL was most efficacious in diminishing the paralysis rate, outperforming RE and its fraction RPF at equivalent concentrations. In contrast, RNF and RWF showed no obvious protective effects. Therefore, we focused our attention on REF for further anti-AD investigations. Subsequent treatments of CL4176 worms with varying concentrations of REF, ranging from 50 μg/mL to 800 μg/mL, showcased that each concentration significantly decreased the paralysis rate, exhibiting a dose-dependent nature (Figure 2C,D). Furthermore, REF demonstrated its capability to diminish the paralysis rate in CL4176 worms at both 33 h and 36 h after a temperature shift to 25 °C (Figure 2E,F). Since Aβ accumulation can cause muscle deterioration, we assessed the physiological effects by measuring pharyngeal pumping and body bending in REF-treated CL4176 worms over a 20 s duration. Our findings reveal that REF treatment led to notable improvements in both pharyngeal pumping and mobility, as compared to untreated worms (Figure 2G,H), suggesting its efficacy in alleviating Aβ-induced behavioral deficits in *C. elegans*. To corroborate the protective effects of REF against Aβ-induced toxicity, we utilized another transgenic strain, CL2006. In this strain, the expression of human Aβ_(1–42)_ protein in body-wall muscle cells is constitutive [18]. Our paralysis assay revealed that REF supplementation decelerated the paralysis progression in CL2006 worms (Figure 2I). Additionally, we investigated the potential of REF in mitigating neurotoxicity induced by abnormal pTau protein, a primary component of neurofibrillary tangles and a hallmark of AD pathology. Using the transgenic BR5270 *C. elegans* strain, which expresses human Tau protein pan-neuronally [21], we found that BR5270 worms had deficits in their food-searching abilities compared to the control strain BR5271, as evidenced by the attenuation of slowing rates. However, REF supplementation significantly neutralized this decline in food-searching capabilities (Figure 2J). Together, our findings indicate that REF possesses the ability to mitigate both Aβ-induced paralysis and Tau-induced neurotoxicity in *C. elegans*.

### 2.3. REF Diminishes the Accumulation of Aβ and pTau Aggregates in C. elegans

**Figure 3:**
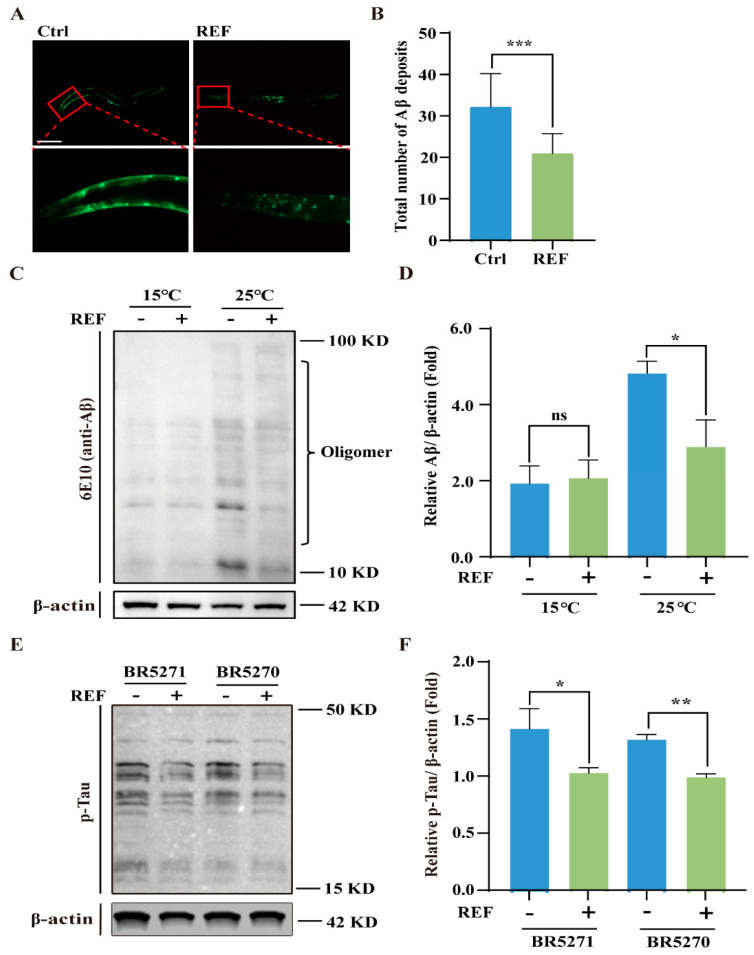
REF enhances degradation of Aβ and pTau in *C. elegans* models of AD. (**A**,**B**) Representative fluorescent images display Aβ deposits in CL2331 worms. Red arrows mark Aβ deposits. Scale bars: 200 μm. Bar graph represents total Aβ deposits. Data: mean ± SD (n = 31 nematodes per group), *** *p* < 0.001. (**C**,**D**) Western blotting image indicates Aβ protein level in CL4176 worms. Bar graph quantifies Aβ protein. Data: mean ± SD (n = 3 independent experiments), ns *p* > 0.05, * *p* < 0.05. (**E**,**F**) Western blotting images show pTau protein level in BR5271 and BR5270 worms. Bar graph quantifies pTau protein. Data: mean ± SD (n = 3 independent experiments), * *p* < 0.05, ** *p* < 0.01. The full-length Western blotting images are shown in Figure S2.

Recent studies have highlighted that the accumulation of Aβ aggregates in the brain acts as a precursor to AD development, often appearing 15–20 years prior to the onset of noticeable clinical symptoms [22]. We set out to explore the effect of REF on Aβ accumulation in *C. elegans*. To begin with, we utilized the CL2331 strain, which expresses the human Aβ_(3–42)_ protein fused with GFP in a temperature-sensitive manner in their body-wall muscle cells [23]. Fluorescence analysis revealed that the quantity of Aβ aggregates was significantly reduced in REF-treated CL2331 worms compared to untreated controls (Figure 3A,B), indicating that REF effectively hinders the formation of Aβ aggregates in vivo. To substantiate this inhibitory effect, we assessed Aβ oligomer levels in the CL4176 strain through Western blotting, using the 6E-10 antibody. Our findings revealed that, although no apparent Aβ oligomers were present in CL4176 worms cultured at 15 °C, a shift to 25 °C resulted in a proliferation of oligomers. Notably, REF supplementation led to a substantial decrease in these oligomers (Figure 3C,D). Furthermore, Western blot analysis demonstrated that REF effectively reduced pTau protein levels in the both BR5271 and BR5270 strains, as evidenced by a decrease in protein intensity (Figure 3E,F). Collectively, these results affirm that REF mitigates the accumulation of both Aβ and pTau aggregates in *C. elegans*.

### 2.4. REF Mitigates Oxidative Stress Triggered by Aβ and Tau Aggregates in C. elegans

**Figure 4:**
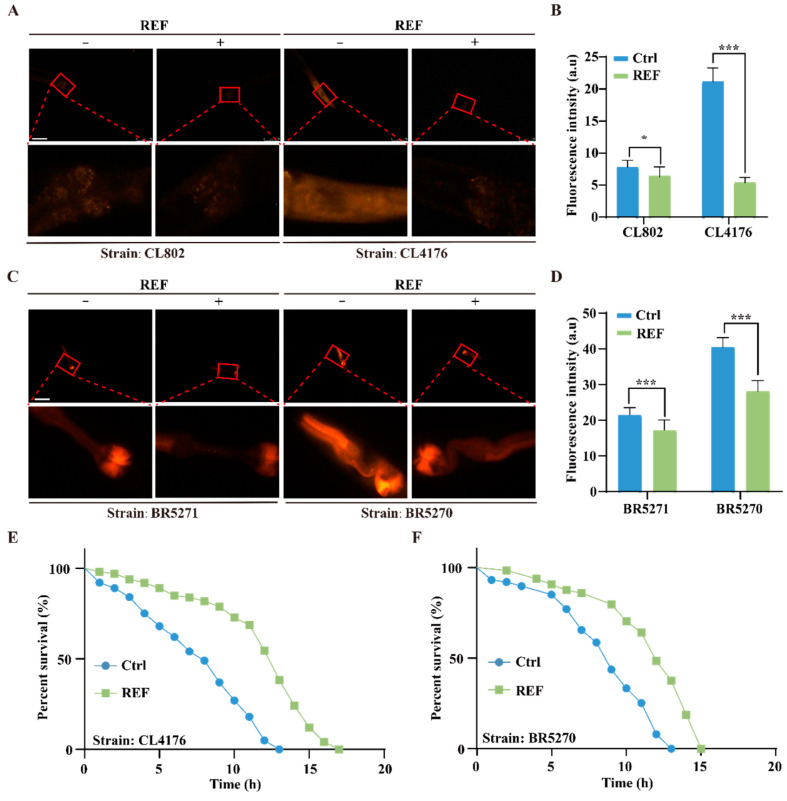
REF alleviates oxidative stress induced by Aβ and Tau aggregates in *C. elegans*. (**A**,**B**) Representative fluorescent images and bar graph illustrate ROS accumulation in CL4176 and control worms CL802 stained with DHE. Data: mean ± SD (n = 15 nematodes per group), * *p* < 0.05, *** *p* < 0.001. Scale bars: 75 μm. (**C**,**D**) Representative fluorescent images and bar graph indicate ROS accumulation in BR5270 and BR5271 worms stained with DHE. Data: mean ± SD (n = 15 nematodes per group), *** *p* < 0.001. Scale bars: 75 μm. (**E**,**F**) Survival curve of H_2_O_2_-treated CL4176 and BR5270 worms in the presence or absence of REF.

Ample research implicates abnormal Aβ and Tau aggregates in mitochondrial dysfunction, which in turn leads to oxidative stress and neuronal cell death in AD patients [24]. To assess the ability of REF to counter this, we employed the dihydroethidium (DHE) staining method to measure ROS levels generated from Aβ and Tau aggregates. Figure 4A,B depicts that DHE fluorescence intensity, an indicator of ROS levels, was elevated in CL4176 worms compared to the wild-type CL802 strain, hinting at ROS generation due to Aβ aggregation. However, REF treatment markedly diminished ROS levels in both CL4176 and CL802 worms. Likewise, REF administration markedly reduced ROS production initiated by Tau proteins in the BR5270 strain and also reduced endogenous ROS levels in the control BR5271 strain (Figure 4C,D). To further examine REF’s role in enhancing oxidative stress resilience, we pre-treated transgenic Aβ or Tau *C. elegans* models with REF and then exposed them to acute oxidative stress induced by 50 mM H_2_O_2_. As depicted in Figure 4E,F, REF treatment substantially increased the survival rates of both CL4176 and BR5270 worms, extending their lifespans by up to 23.07% and 15.38%, respectively. Together, our findings establish that REF effectively mitigates oxidative stress induced by Aβ and Tau aggregates in *C. elegans*.

### 2.5. REF Activates Autophagy in C. elegans and U87 Cells

**Figure 5:**
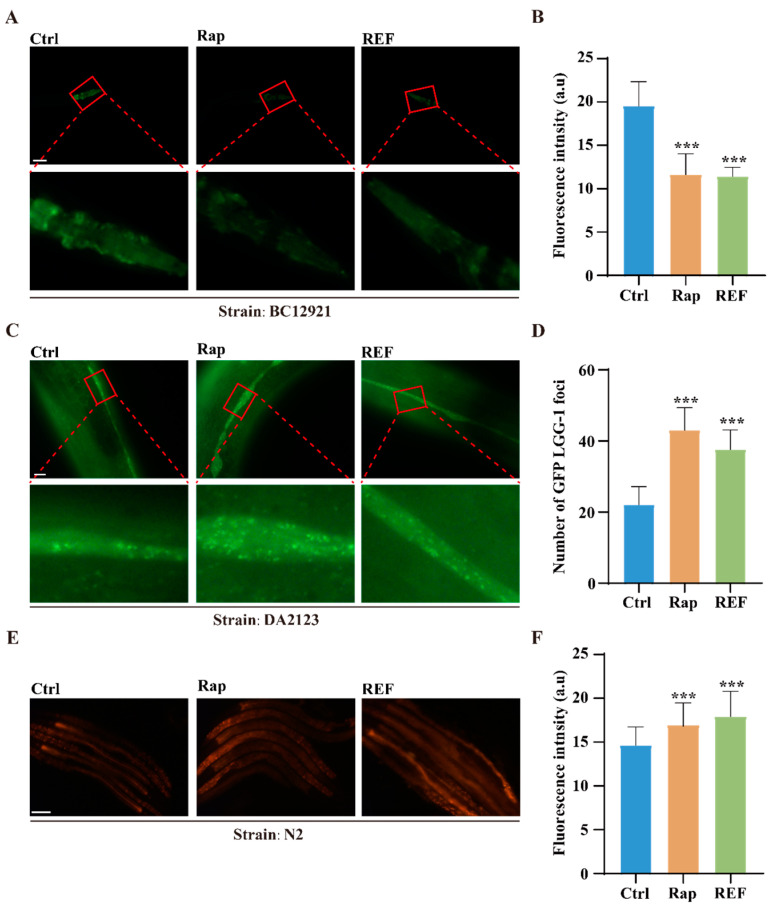
REF induces autophagy in *C. elegans*. (**A**,**B**) Representative fluorescent images display SQST-1::GFP expression in BC12921 worms. Bar graph quantifies GFP levels. Data: mean ± SD (n = 20 nematodes per group), *** *p* < 0.001. Scale bars: 50 μm. (**C**,**D**) Representative fluorescent images show autophagosomes (GFP::LGG-1 puncta) in seam cells of DA2123 worms. Bar graph quantifies GFP::LGG-1 puncta. Data: mean ± SD (n = 17 nematodes per group), *** *p* < 0.001. Scale bars: 10 μm. (**E**,**F**) Lysotracker staining images of REF-treated N2 worms. Bar graph quantifies GFP intensity. Data: mean ± SD (n = 40 nematodes per group), *** *p* < 0.001. Scale bars: 100 μm.

Our above findings have indicated that REF effectively suppresses the accumulation of Aβ and pTau aggregates in *C. elegans*. With the rising understanding of autophagy’s essential role—as a primary proteolytic mechanism—in breaking down misfolded proteins such as Aβ and pTau [25], we set out to determine if REF can stimulate autophagy in *C. elegans*. For this investigation, we used two popular transgenic *C. elegans* models, BC12921 and DA2123. The BC12921 strain expresses a p62/SQST-1-GFP fusion protein, which becomes degraded during heightened autophagy [26]. Through fluorescence assays, we observed a significant decrease—over 41.73%—in GFP intensity (representing p62 protein levels) in REF-treated BC12921 worms compared to their untreated counterparts (Figure 5A,B). In contrast, the DA2123 strain produces an LGG-1-GFP fusion protein, which associates with the autophagosome membrane and forms puncta during autophagy activation [26]. Our observations showed that, while untreated DA2123 worms displayed diffuse cytoplasmic fluorescence across various tissues, REF treatment led to the formation of LGG-1^+^ puncta specifically in their seam cells (Figure 5C,D), which is indicative of autophagy activation. Furthering our research, we conducted lysotracker staining on REF-treated N2 worms. Lysotracker serves as an indicator for lysosomes and late endosomes; increased staining usually points to elevated levels of these organelles, often a sign of enhanced autophagic activity. Our results showed that REF treatment significantly increased lysotracker staining in N2 worms (Figure 5E,F), implying an upregulation of autophagic activity. Additionally, in stable RFP-GFP-LC3 U87 cells, we ascertained the autophagy-inducing effects of REF. Notably, REF significantly amplified GFP-LC3 puncta in these cells (Figure S1). Together, our collective findings confirm REF’s capacity to stimulate autophagy, both in *C. elegans* and U87 cells.

### 2.6. REF Alleviates Aβ- and Tau-Induced Neurotoxicity via Autophagy Activation in C. elegans

**Figure 6:**
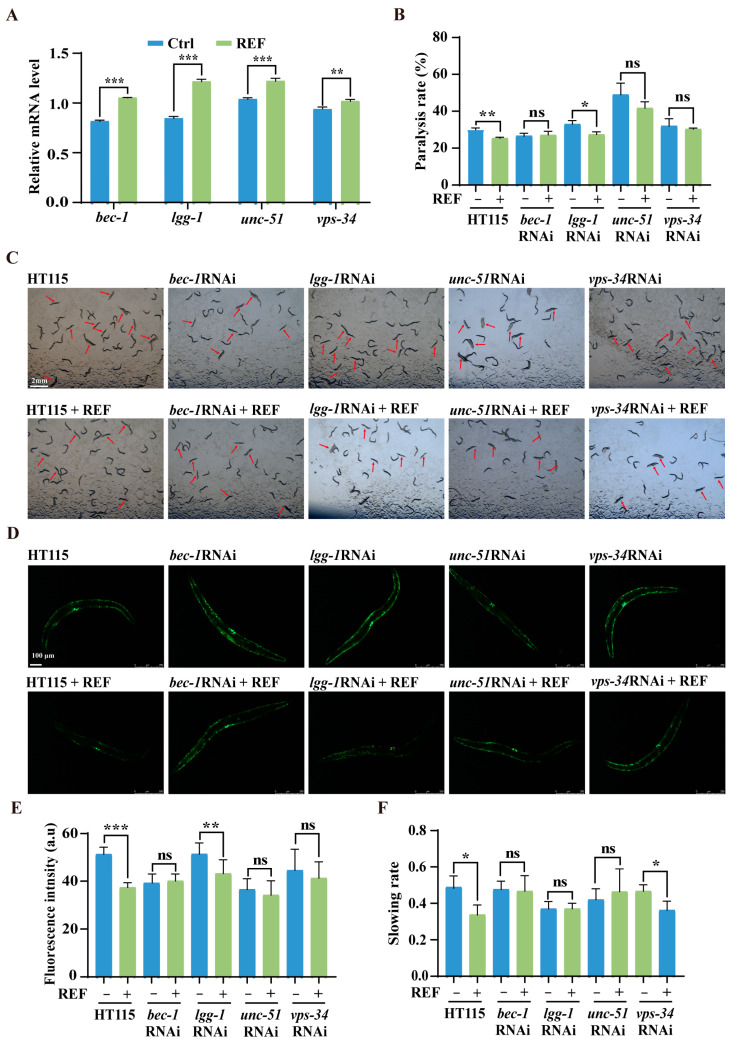
REF mitigates Aβ- and Tau-mediated pathology via autophagy genes in *C. elegans*. (**A**) qRT-PCR detects autophagy-related genes (*bec-1*, *lgg-1*, *unc-51*, and *vps-34*) in N2 worms treated with REF. Data: mean ± SEM (n = 3 independent experiments), ** *p* < 0.01, *** *p* < 0.001. (**B**,**C**) Representative images show the paralyzed CL4176 worms fed with RNAi bacteria from *bec-1*, *lgg-1*, *unc-51*, and *vps-34*. Scale bars: 2 mm. Bar graph indicates the paralysis rate of worms. Data: mean ± SD (n = 3 independent experiments), ns *p* > 0.05, * *p* < 0.05, ** *p* < 0.01. (**D**,**E**) Representative fluorescent images reveal Aβ deposits in CL2331 worms fed with RNAi bacteria from *bec-1*, *lgg-1*, *unc-51*, and *vps-34*. Scale bars: 100 μm. Bar graph quantifies total Aβ deposits. Data: mean ± SD (n = 15 nematodes per group), ns *p* > 0.05, ** *p* < 0.01, *** *p* < 0.001. (**F**) Bar graph displays slowing rate of BR5270 worms fed with RNAi bacteria of *bec-1*, *lgg-1*, *unc-51*, and *vps-34*. Data: mean ± SD (n = 3 independent experiments), ns *p* > 0.05, * *p* < 0.05.

Given the autophagy-activating capabilities of REF in *C. elegans*, we sought to understand whether this mechanism could neutralize the toxic effects of Aβ and Tau. We began by evaluating mRNA expression levels of pivotal autophagy genes via qRT-PCR assay. As illustrated in Figure 6A, REF considerably elevated the expression of key autophagy genes, namely *bec-1*, *lgg-1*, *unc-51*, and *vps-34*, hinting at their potential involvement in REF-mediated autophagy enhancement. To further elucidate this relationship, we employed RNAi, a widely recognized gene silencing approach in *C. elegans*, to suppress the four autophagy genes elevated by REF. Subsequently, we examined the toxicity triggered by Aβ and Tau in *C. elegans* AD models. Our findings indicated that REF treatment significantly reduced the paralysis rate in CL4176 worms when exposed to the control *E.coli* HT115 strain. However, this reduction was notably hampered when the worms ingested RNAi bacteria-inhibiting *bec-1*, *unc-51*, and *vps-34* genes (Figure 6B,C). Additionally, the suppression of Aβ aggregate accumulation by REF was also nullified under these conditions (Figure 6D,E). In contrast, the knockdown of *lgg-1* did not compromise REF’s effectiveness against Aβ-induced toxicity, suggesting that this gene may not be critical for REF’s protective mechanisms (Figure 6B–E). In parallel, our experiments with the BR5270 worms showed that the RNAi-mediated suppression of key autophagy genes *bec-1*, *lgg-1*, and *unc-51*, except for *vps-34*, negated the improvements that REF rendered against Tau-associated behavioral defects (Figure 6F). Collectively, our results offer compelling evidence that the protective effects conferred by REF against Aβ- and Tau-mediated neurotoxicity are intrinsically tied to the autophagy pathway in *C. elegans*.

## 3. Discussion

Prior investigations have illuminated multiple pharmacological attributes of *R. carnea* [10,11,12], yet its prospective anti-AD efficacy has largely remained unexplored. In this research, we initially concentrated on the *R. carnea* ether fraction (REF), as it exhibited the most significant anti-paralysis effects compared to RE and other fractions (RNF, RPF, and RWF) in the *C. elegans* AD model of CL4176. Further exploration revealed the pioneering discovery that REF possesses notable protective effects against Aβ- and Tau-induced toxicity in several *C. elegans* AD models. Specifically, REF not only reduced the rate of paralysis in CL4176 worms but also augmented their physiological processes, such as body bending and pharyngeal pumping. Moreover, REF displayed effectiveness in ameliorating the food perception deficits observed in BR5270 worms. Crucially, REF also diminished the accumulation of pathogenic Aβ and pTau protein aggregates while countering the associated oxidative stress. Our mechanistic analyses further elucidated the fact that autophagy serves as a cornerstone in mediating these protective outcomes, amplifying REF’s promise as a candidate for therapeutic interventions in AD and related NDs.

The employment of *C. elegans* as our experimental platform offers a strategic advantage, particularly considering its cost-effective nature and amenability to high-throughput drug screening [27]. While *C. elegans* may not fully mirror the intricate pathophysiology observed in human or higher vertebrate models, its efficacy in drug discovery and early-stage preclinical research is well-established. This organism has been instrumental in various scientific areas, from studying the mechanisms of aging and neurodegeneration to exploring treatments for pathogen infection and metabolic disorders [28]. In light of these attributes, we deployed a multifaceted approach with several transgenic *C. elegans* models to perform a comprehensive evaluation of REF’s anti-AD potential. The findings strongly corroborate REF’s capability to delay the onset of paralysis, diminish the accumulation of harmful protein aggregates, and improve behavioral attributes adversely affected by Aβ and Tau proteins. However, it is crucial to recognize the inherent limitations of *C. elegans* as a model organism, including its relatively simplistic nervous system and the absence of some cellular structures present in higher organisms [28]. Such limitations underline the imperative for further confirmatory research using higher-order animal models, like zebrafish or mice, to fully comprehend the spectrum of REF’s therapeutic capabilities. The next steps would involve a broader spectrum of in vivo studies to establish REF’s efficacy and safety profiles in the context of AD and other NDs.

Plenty of compelling evidence highlights the significance of autophagy, a highly conserved cellular pathway responsible for breaking down and recycling unwanted components, such as defective organelles and misfolded protein aggregates, through lysosomes [29]. This process has gained substantial attention due to its potential role in counteracting aging and ameliorating age-related diseases, particularly NDs. Recent investigations, spanning from simple organisms like *C. elegans* to more complex models like rodents and human cell lines, have shed light on the decline in autophagic activity with advancing age [30]. This decline not only exacerbates cellular dysfunction but also appears to be a precursor to the development of age-related diseases. Moreover, recent research carried out on laboratory animals and human samples has highlighted a decline in the expression of autophagy-related genes such as *Becn1*, *Atg5*, and *Atg7* in the brains of individuals with AD and in AD mouse models [31]. However, boosting autophagy, either genetically or pharmacologically, can ameliorate AD pathologies across various organisms. For instance, augmenting *Beclin-1* expression in *Becn1*^F121A/F121A^ mice has been shown to mitigate memory loss and cognitive decline induced by Aβ and Tau [32]. Similarly, the administration of autophagy-inducing compounds like NAD^+^, Rap, and carbamazepine, which activate autophagy either through mTOR-dependent or -independent pathways, has demonstrated the ability to prevent memory loss and rescue cognitive deficits in both Aβ and Tau *C. elegans* and mouse models of AD [33,34,35]. These discoveries underscore the potential of enhancing autophagy as a therapeutic strategy for managing AD. In our current study, we have effectively validated REF’s capability to enhance autophagy. This validation was achieved through the utilization of *C. elegans* strains BC12921 and DA2123, wherein we observed a clear reduction in p62/SQST-1-GFP levels alongside a notable increase in GFP::LGG-1 puncta following REF treatment. Importantly, the enhanced autophagy-promoting effects of REF were further corroborated in our experiments using RFP-GFP-U87 cell lines, which exhibited an evident rise in GFP-LC3 puncta. Although our study has compellingly established REF’s potential to activate autophagy, there remains a need for deeper exploration in the form of identifying specific autophagy enhancers and elucidating their underlying molecular mechanisms.

In order to ascertain the role of REF-induced autophagy and its neuroprotective effects, we employed RNAi bacteria to suppress the upregulated autophagy genes facilitated by REF in *C. elegans* models of AD. The results observed in CL4176 and BR5270 worms fed with RNAi bacteria that targeted autophagy genes revealed a significant disruption in the neuroprotective effects of REF against AD. This disruption was evident in terms of delayed paralysis and the reversal of food-sensing behavior defects. Nonetheless, comprehending the intricate mechanism through which REF induces autophagy for its anti-AD effects demands further investigation. Our study demonstrated that, while REF upregulated four autophagy-related genes (*bec-1*, *lgg-1*, *unc-51*, and *vps-34*), only the knockdown of *bec-1*, *unc-51*, and *vps-34* substantially attenuated the inhibitory impact of REF on Aβ-mediated paralysis in CL4176 worms. This implies that the positive effects of REF in mitigating Aβ-induced toxicity are largely reliant on these specific autophagy-related genes. Conversely, in *C. elegans* models of Tau-induced AD, the neuroprotective effects of REF against Tau-induced toxicity were found to hinge on *bec-1*, *lgg-1*, and *unc-51*, but not on *vps-34*. These findings underscore the involvement of distinct autophagy-related genes in mediating REF’s beneficial effects on Aβ- and Tau-induced toxicity. For a more comprehensive understanding of REF’s actions, further studies will delve deeper into the underlying mechanisms across various *C. elegans* models of AD.

In summary, our study has effectively identified REF as a promising anti-AD drug, capable of ameliorating Aβ and Tau pathology in *C. elegans* models through autophagy activation. While these findings are promising, further experiments are imperative to uncover the anti-AD effects and autophagy activity of specific active ingredients in REF, such as Coumarin, L(+)-Arginine, Vitamin C, L-Cystathionine, Palmitoleic acid, Genistein, and Dammaradienol. This deeper understanding will enhance its potential for therapeutic applications in age-related diseases like AD.

## 4. Methods and Materials

### 4.1. Extraction and Fractionation of R. carnea

*R. carnea* was supplied and authenticated by Yongyi TCM Co., Ltd. (Anguo, China). Approximately 100 g of the herb were finely chopped and ground into a powder with a particle size between 40 and 60 mesh. This powder was subjected to three rounds of extraction using 500 mL of 95% methanol, each lasting for 2.5 h at 65 °C. The extracts from all three rounds were combined, filtered, and concentrated under vacuum using a rotary evaporator. For the filtration process, a vacuum filtration system was utilized. A ceramic Büchner funnel (Abooly, 100 mm) was fitted with wet filter paper (Whatman No. 1, 9 cm) and positioned atop a filtration flask (Schlenk, 1000 mL). The circulating water vacuum pump (SHZ-D(III), China) was then started to facilitate the filtration process. The resultant residue, referred to as RE, was collected. RE was then reconstituted in water and partitioned successively using petroleum ether, ethyl acetate, and n-butanol to yield the petroleum ether fraction (RPF), REF, n-butanol fraction (RNF), and water fraction (RWF), respectively. The fractionation process was based on solvent polarity, allowing for the separation of components with varying polarities.

### 4.2. UPLC/Q-TOF-MS/MS Conditions

Sample analyses were conducted using a Shimadzu UHPLC system, comprising the LC-3AD solvent delivery system, SIL30ACXR autosampler, CTO-30AC column oven, DGU-20A3 degasser, and CBM-20A controller. The separation process utilized an Agilent Zorbax EcLipse Plus C18 column within the UHPLC system, with a flow rate of 0.3 mL/min (dimensions: 1.8 mm × 100 mm × 2.1 mm) while maintaining the column temperature at 40 °C. The mobile phase consisted of water with 0.1% formic acid (A) and acetonitrile with 0.1% formic acid (B). The elution program was as follows: 0–18 min, 5–95% B; 18.01–20 min, 5% B. For mass spectrometry analysis, a triple TOFM X500R system equipped with a Duo Spray source from AB SCIEX in Foster City, CA, USA, was employed in both negative and positive electrospray ionization modes. In the negative ESI mode, the ion spray voltage was set at −4500, curtain gas at 25 psi, ion source temperature at 0 °C, declustering potential (DP) at −80 V, nebulizer gas (GS 1) at 20 psi, and heater gas (GS 2) at 0 psi. The mass scan ranged from m/z 100 to 1000 Da. In the positive ESI mode, the ion spray voltage was 5500, curtain gas was 25 psi, ion source temperature was 0 °C, DP was 50 V, GS 1 was 20 psi, and GS 2 was 0 psi. The mass scan also covered m/z 100–1000 Da. Data analysis was performed using Peak View^®^ 1.4 software, provided by AB SCIEX in Foster City, CA, USA.

### 4.3. Maintenance and Synchronization of C. elegans Strains

All *C. elegans* strains utilized in this study were obtained from the Caenorhabditis Genetics Center (CGC, University of Minnesota, Minneapolis, MN, USA). A comprehensive list of strain details is available in Table S1. The worms were cultured at 20 °C on nematode growth medium (NGM) agar plates and fed live *E. coli* (OP50) as their food source. For synchronization, gravid worms were treated with a bleaching solution comprising 0.5 M NaOH and 1% NaClO. The eggs were then allowed to hatch in M9 buffer overnight at 20 °C. Upon reaching the L4 larval stage, the worms were transferred to new NGM plates containing 5 mg/L 5-fluoro-2′-deoxyuridine (FUDR, Aladdin) to arrest further egg development.

### 4.4. Paralysis Assay

Two transgenic *C. elegans* strains, CL4176 and CL2006, were utilized to assess the paralysis-inducing effects of Aβ_(1–42)_. For CL4176, worms were initially incubated at 16 °C for 36 h on NGM plates, with or without REF. Aβ expression and aggregation were induced by elevating the incubation temperature to 25 °C for an additional 36 h. Paralysis was evaluated as described in previous studies [36]. For CL2006, synchronized larvae at the late L4 or early adult stage were moved to NGM/FUDR plates, in the presence or absence of REF. Paralysis was assessed on the 5th day post-adulthood. A minimum of 60 worms was included in each experimental group to ensure statistical validity.

### 4.5. Measurement of Aβ_(3–42)_ Aggregation in CL2331 Worms

The aggregation of Aβ was measured using the CL2331 worm strain, which expresses GFP-Aβ_(3–42)_ in its body-wall muscle in a temperature-sensitive manner. Briefly, CL2331 worms, both REF treated and untreated, were shifted from 15 °C to 23 °C until they reached adulthood to induce Aβ_(3–42)_ aggregation. Subsequently, these worms were immobilized on glass slides and imaged using a fluorescence microscope (Leica DM6B, Leica Microsystems GmbH, Wetzlar, Germany). Fluorescent images were analyzed, specifically focusing on Aβ deposits in the anterior regions of the worms. A sample size of at least 25 worms per experimental group was utilized for statistical analysis.

### 4.6. Western Blot Analysis for Aβ and pTau Levels and Antibodies

CL4176 worms, after being incubated at 15 °C or 25 °C for 36 h with or without REF treatment, were harvested. Similarly, BR5270 and BR5271 worms were collected after a 48 h treatment period with or without REF. Worm samples were lysed in 1.5 mL tubes containing 100 μL of CST lysis buffer with protease inhibitors. Following three 15 s sonication cycles, the lysates were centrifuged at 15,000 rpm for 15 min at 4 °C. The supernatant protein concentration was determined, and equal protein quantities were denatured at 99 °C for 5 min and then subjected to SDS-PAGE separation on 12.5% polyacrylamide gels. Electrophoresis was carried out at 80 volts for 30 min, followed by 120 volts for 1 h. The separated proteins on the SDS-PAGE gel were then transferred onto a polyvinylidene fluoride (PVDF) membrane at 250 mA for 2 h. Subsequently, the PVDF membrane was blocked with 5% non-fat milk for an hour, and incubated with primary antibodies overnight at 4 °C. This was followed by a one-hour incubation with secondary antibodies at room temperature. Post TBST wash, protein bands were visualized using ECL reagents (4A Biotech, Beijing, China) and captured with a ChemiDoc™ Imaging System. Band intensities were quantified using ImageJ software version 1.52a (National Institutes of Health, Bethesda, MD, USA). To detect the accumulation of Aβ species in CL4176 worms, the anti-Aβ antibody (6E10, #SIG-39320) from Biolegend Inc. (San Diego, CA, USA) was employed, following established protocols from prior studies [37]. In the investigation of pTau aggregate accumulation in transgenic BR5270 and BR5271 *C. elegans*, we utilized pTau (Ser404, #20194s) from Cell Signaling Technologies Inc. (CST, Beverly, MA, USA) in accordance with methodologies outlined in a recent study [38]. β-actin antibodies (#66009-1-Ig) were obtained from Proteintech Group, Inc. (Rosemont, IL, USA).

### 4.7. Assessment of Food-Sensing Behavior

The BR5270 worms, which express the pro-aggregant F3ΔK280 human Tau, along with its vector control BR5271, were used to assess the influence of REF on enhancing food-sensing behavior in worms. Briefly, both sets of worms, treated with REF and untreated, were grown on NGM plates for 48 h. Following this, the worms were rinsed, collected with M9 buffer, and cultured on new NGM agar plates, either with or without the *E. coli* OP50 strain. After allowing them to adapt for 5 min, the frequency of body bends was counted at intervals of 20 s. The slowing rate, indicative of the worms’ food-sensing behavior, was calculated as previously described in the methodology of [38]. Statistical analysis required a minimum sample size of 15 worms per group.

### 4.8. Assessment of ROS Levels and Oxidative Stress in C. elegans

To determine the inhibitory effect of REF on ROS triggered by Aβ and Tau, we used the transgenic *C. elegans* strains CL4176 and BR5270. These worms were stained with a 100 μM DHE solution in a total volume of 500 μL, and incubated for one hour at 20 °C. Following this, 10 mM sodium azide was applied to immobilize the worms, which were then mounted on a 2% agarose pad on a glass slide. Fluorescence microscopy was employed to capture images of the stained worms. ImageJ software version 1.52a was utilized to measure the intensity of the red fluorescence, serving as an indicator of intracellular ROS levels. To assess the potential of REF to confer oxidative stress resistance, L1-stage CL4176 or BR5270 worms were moved to NGM plates containing 100 μg/mL of REF. These plates were incubated at 20 °C for a period of 48 h. Then, the treated worms were transferred to fresh NGM plates containing 50 mM H_2_O_2_, both with and without the presence of REF. Mortality rates were recorded at hourly intervals until all worms were accounted for. Each of these assays was conducted in triplicate, with each replicate containing a minimum of 60 worms for evaluation.

### 4.9. Gene Silencing through RNAi

An RNAi library specifically for *C. elegans* was procured from Horizon Discovery (Catalog number: OCE1182), which was originally constructed by Julie Ahringer’s group. Gene silencing was achieved by feeding the worms bacteria that produce RNA against target genes, as described in previous studies [26]. Briefly, RNAi and HT115 bacteria were grown in LB medium containing 50 µg/mL ampicillin at 37 °C overnight. Following this, 5 mM isoprophylthiogalactoside (IPTG) was introduced to facilitate double-stranded RNA synthesis. Synchronized L1 worms, either treated with REF or left untreated, were then fed with RNAi targeting genes linked to autophagy, including *bec-1*, *lgg-1*, *unc-51*, and *vps-34*. The interference effect of the target genes in *C. elegans* has been identified in our previous studies [39].

### 4.10. RNA Isolation and Quantitative Real-Time PCR (qRT-PCR)

Synchronized L1 worms, cultivated on NMG plates with or without REF, were harvested upon reaching the late L4 larvae or early adult stage. The collected worms were washed with M9 buffer, and RNA was extracted using TRIzol Reagent after snap freezing in liquid nitrogen. RNA purification included two chloroform-based separation steps, an isopropanol precipitation, and a final wash with 70% ethanol. Extracted RNA was reconstituted in RNase-free water. For cDNA synthesis, 1 μg of total RNA was reverse transcribed using the One-Step cDNA Synthesis Kit (TSK301S, Tsingke Biotechnology Co., Ltd., Beijing, China). qRT-PCR reactions, with SYBR Green PCR Master Mix (TSE201, Tsingke Biotechnology Co., Ltd., Beijing, China), were carried out on a Bio-Rad CFX96 Real-Time System attached to a C1000 Thermal Cycler (Bio-Rad, Hercules, CA, USA). Transcript levels were normalized to *cdc42*. A comprehensive list of primers employed is available in Table S2.

### 4.11. Data Analysis and Statistics

Statistical analyses were performed on data collected from at least three separate experiments. For evaluating paralysis frequency and resistance to stress, Kaplan–Meier survival curves were generated. The log-rank test was utilized to calculate *p*-values in these contexts. All other data are presented as the mean value accompanied by the standard deviation (SD). For inferential statistics, one-way Analysis of Variance (ANOVA) followed by Tukey’s post-hoc test and two-way ANOVA were applied as appropriate. The statistical software GraphPad Prism version 8.01 (San Diego, CA, USA) was employed for all data analyses. A *p*-value of less than 0.05 was deemed to be indicative of statistical significance.

## Figures and Tables

**Table 1:** The chemical name, retention time, chemical formula, mass, and distribution of the identified potential components in *R. carnea*.

Chemical Name	Retention Time (min)	Chemical Formula	Expected Mass (Da)	Observed Mass (Da)	Mass Error (Da)	Distribution
Coumarin	5.23	C_9_H_6_O_2_	181.0121	181.0162	0.0041	REF
L(+)-Arginine	0.84	C_6_H_14_N_4_O_2_	175.1159	175.1167	0.0008	
Vitamin C	1.86	C_6_H_8_O_6_	175.021	175.0262	0.0052	
L-Cystathionine	4.18	C_7_H_14_N_2_O_4_S	257.0379	257.0311	−0.0068	
Palmitoleic acid	11.16	C_16_H_30_O_2_	277.2131	277.2162	0.0031	
Genistein	7.98	C_15_H_10_O_5_	269.0423	269.0479	0.0056	
Dammaradienol	17.67	C_30_H_50_O	427.3741	427.3784	0.0043	
Cianidanol	1.74	C_15_H_14_O_6_	289.0651	289.0682	0.0031	RNF
Lauric acid	10.77	C_12_H_24_O_2_	223.1639	223.1691	0.0052	RPF
Palmitic acid	13.42	C_16_H_32_O_2_	279.229	279.2282	−0.0008	
Thymidine	1.78	C_10_H_14_N_2_O_5_	277.0576	277.0587	0.0011	RWF
D-Erythro-Sphingosine	12.93	C_18_H_37_NO_2_	300.2855	300.2897	0.0042	
Gibberellin A38	9.57	C_20_H_26_O_6_	397.1512	397.1512	0	
D-Xylulose	1.06	C_5_H_10_O_5_	149.0427	149.0473	0.0046	RE
Guanine	1.78	C_5_H_5_N_5_O	152.0598	152.0571	−0.0027	
Guanosine	1.78	C_10_H_13_N_5_O_5_	284.1027	284.1005	−0.0022	
Gibberellin A64	8.97	C_20_H_26_O_5_	345.1794	345.1732	−0.0062	
D-beta-Tocotrienol	9.64	C_28_H_42_O_2_	433.3259	433.3241	−0.0018	
[12]-Gingerol	7.59	C_23_H_38_O_4_	401.2617	401.2614	−0.0003	
Torvoside A	8.46	C_45_H_76_O_18_	903.4658	903.4636	−0.0022	
DL-2-Aminoadipic aicd	1.63	C_6_H_11_NO_4_	184.0653	184.0609	−0.0044	REF, RE
D-erythro-C18-Dihydro-D-sphingosine	15.7	C_18_H_39_NO_2_	324.2835	324.2899	0.0064	
2″-O-alpha-L-Rhamnopyranoside-Orientin	6.23	C_27_H_30_O_15_	595.1647	595.1658	0.0011	RWF, RE
Adenosine	1.55	C_10_H_13_N_5_O_4_	268.1022	268.1044	0.0022	
Ganoderenic acid E	9.7	C_30_H_40_O_8_	527.2629	527.2688	0.0059	RNF, RE
Capsicoside C2	9.12	C_44_H_72_O_17_	907.4485	907.4495	0.001	
trans-Traumatic acid	7.98	C_12_H_20_O_4_	229.1411	229.1435	0.0024	REF, RPF
(R)-(−)-Citramalic acid	2.69	C_5_H_8_O_5_	171.0253	171.0291	0.0038	REF, RWF
Gallic acid	4.65	C_7_H_6_O_5_	169.0133	169.0161	0.0028	
Homovanillic acid	2.07	C_9_H_10_O_4_	181.0514	181.0523	0.0009	
Linolenic acid	12.22	C_18_H_30_O_2_	277.2133	277.2185	0.0052	RWF, RPF
L-Valine	0.95	C_5_H_11_NO_2_	118.0857	118.0857	0	RWF, RNF, RE
Solasodine	17.32	C_27_H_43_NO_2_	414.3526	414.3575	0.0049	REF, RPF, RE
Decanoic acid	9.74	C_10_H_2_0O_2_	195.1356	195.1384	0.0028	
Phytosphingosine	10.94	C_18_H_39_NO_3_	318.2969	318.2999	0.003	REF, RPF, RWF
pyridoxal	5.35	C_8_H_9_NO_3_	190.0485	190.0499	0.0014	REF, RNF, RE
L-Phenylalanine	1.86	C_9_H_11_NO_2_	200.0547	200.0582	0.0035	
Tryptamine	6.6	C_10_H_12_N_2_	183.0917	183.0915	−0.0002	
L-Leucine	0.96	C_6_H_13_NO_2_	132.101	132.1016	0.0006	RWF, RNF, REF
Allantoic acid	5.2	C_4_H_8_N_4_O_4_	177.0534	177.0533	−0.0001	
L-Malic acid	0.94	C_4_H_6_O_5_	133.013	133.015	0.002	REF, RNF, RWF, RE
Salicylic acid	3.16	C_7_H_6_O_3_	137.0233	137.0261	0.0028	
Inositol	0.92	C_6_H_12_O_6_	179.0533	179.0579	0.0046	
L-Citrulline	0.93	C_6_H_13_N_3_O_3_	198.0913	198.0979	0.0066	RPF, REF, RNF, RWF
Hexadecanamide	16.83	C_16_H_33_NO	256.2602	256.2634	0.0032	RWF, RPF, RNF, RE

## Data Availability

All of the figures used to support the findings of this study are included within the article.

## Supplementary Materials

The following supporting information can be downloaded at: https://www.mdpi.com/article/10.3390/ijms242216536/s1.

## Abbreviations

AD	Alzheimer’s disease
Aβ	β-amyloid
pTau	Hyperphosphorylated Tau
TCMs	Traditional Chinese medicines
*C. elegans*	Caenorhabditis elegans
NDs	Neurodegenerative diseases
RE	*R. carnea* methanol extract
REF	*R. carnea* ether fraction
RNAi	RNA interference
CGC	Caenorhabditis Genetics Center
NGM	Nematode growth medium
FUDR	5-fluoro-2′-deoxyuridine
IPTG	Isoprophylthiogalactoside
qRT-PCR	Quantitative Real-Time PCR
SD	Standard deviation
DHE	Dihydroethidium
