# Supplementary material for: The Protective Effects of Reineckia carnea Ether Fraction against Alzheimer’s Disease Pathology: An Exploration in Caenorhabditis elegans Models
Source: Int J Mol Sci. 2023 Nov 20;24(22):16536. doi: 10.3390/ijms242216536 (PMC10672099; doi:10.3390/ijms242216536)
Supplement: Supplementary file 1 [file ijms-24-16536-s001.zip › ijms-2700242-supplementary.pdf]

Supplemental Material

Supplemental Figures

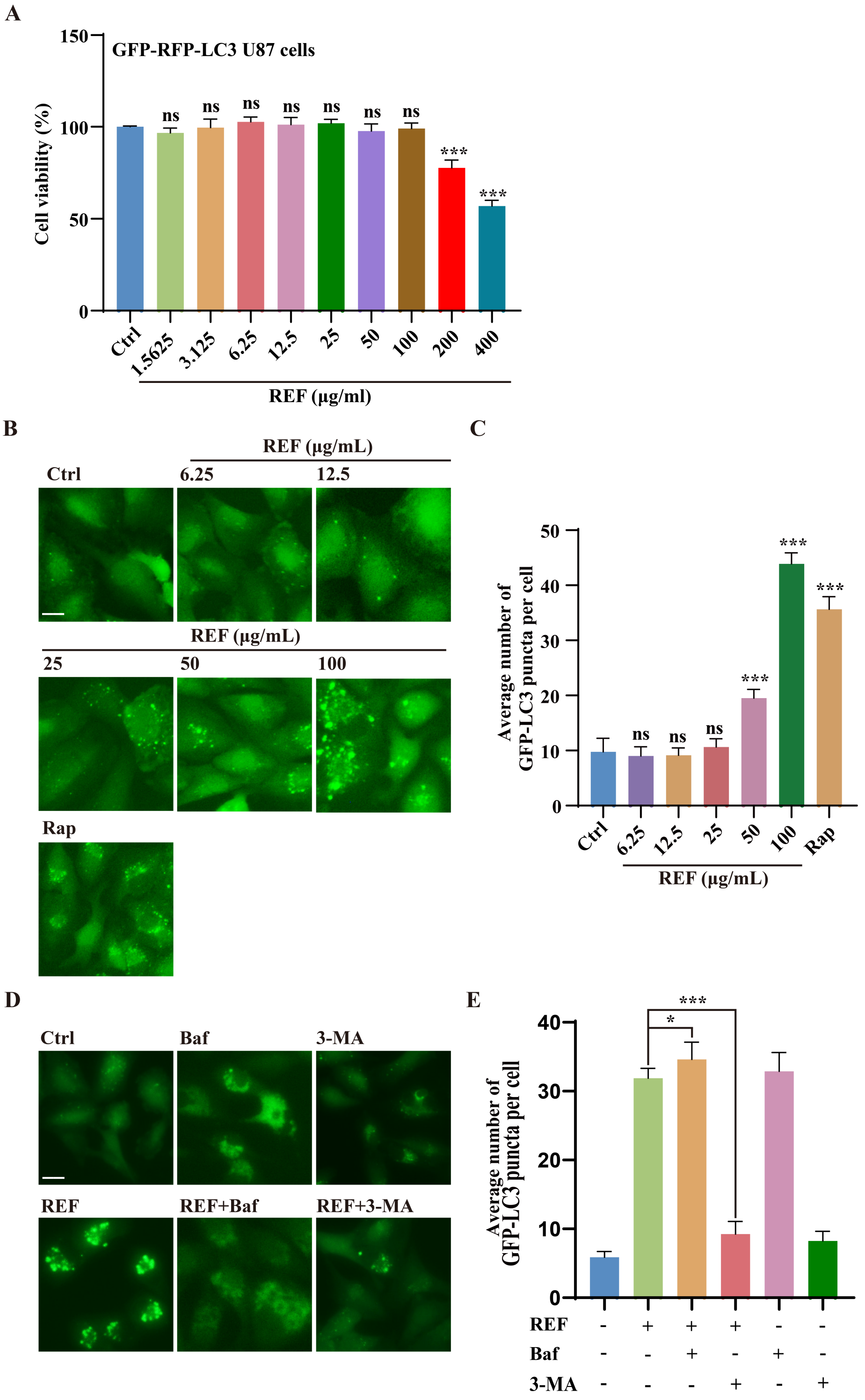

Figure S1. REF induces autophagy in U87 cells. (A) Bar graph indicates the viability of GFP-RFP-LC3 U87 cells treated with different concentrations of REF. Data: mean

± SD (n= 3 independent experiments), ns $p > 0.05$, *** $p < 0.001$. **(B-C)** Representative fluorescent images indicate GFP-LC3 puncta in GFP-RFP-LC3 U87 cells treated with different concentrations of REF. Scale bars: 50 μm. Bar graph indicates the number of GFP-LC3 puncta in GFP-positive cells. Data: mean ± SD (n= 3 independent experiments), ns $p > 0.05$, *** $p < 0.001$. **(D-E)** Representative fluorescent images indicate GFP-LC3 puncta in GFP-RFP-LC3 U87 cells treated with autophagy inhibitors 3-MA and Baf in the presence or absence of REF. Scale bars: 50 μm. Bar graph indicates the number of GFP-LC3 puncta in GFP-positive cells. Data: mean ± SD (n= 3 independent experiments), * $p < 0.05$, *** $p < 0.001$.

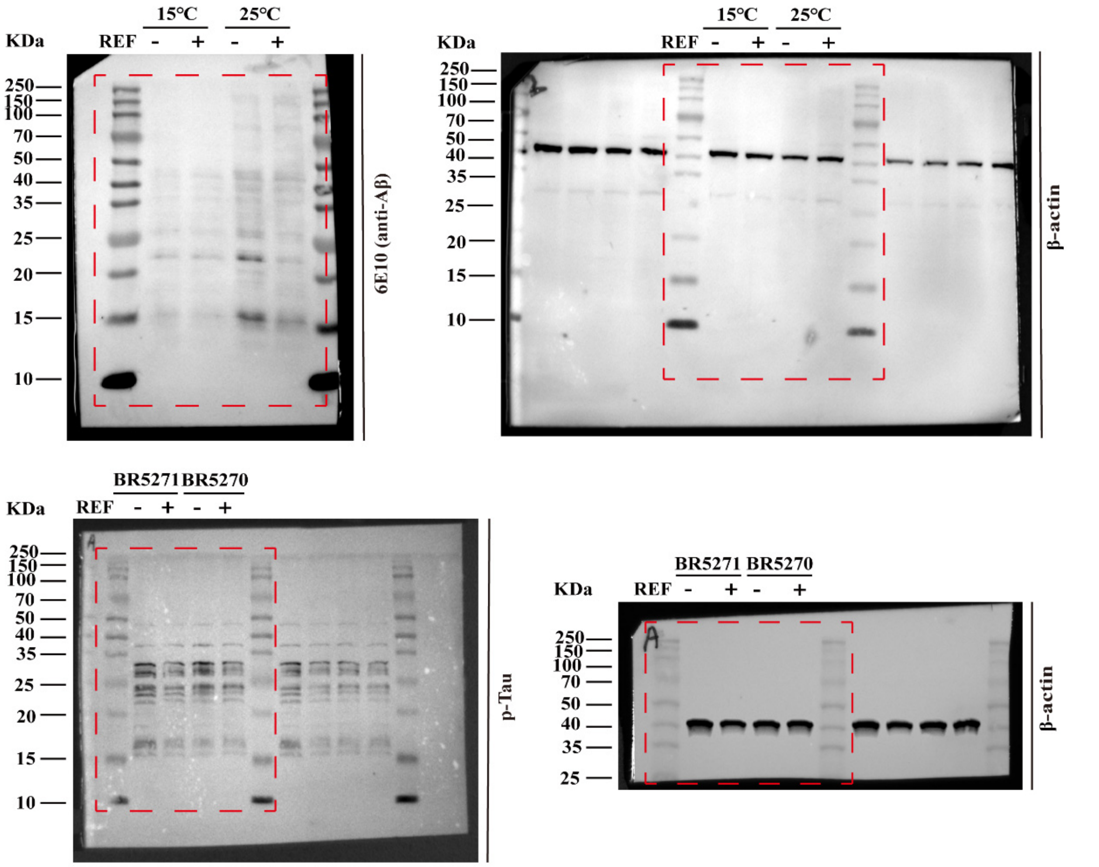

Figure S2. Full-length Western blotting images of Fig. 3.

Supplemental Tables

Table S1. Information on the *C. elegans* strains used in this study.

Strain	Descriptions	Website
N2	Wild-type. *C. elegans* var Bristol	https://cgc.umn.edu/strain/N2
CL4176	dvIs27 [myo-3p::A-Beta (1-42)::let-851 3'UTR) + rol-6(su1006)] X	https://cgc.umn.edu/strain/CL4176
CL2006	vIs2 [pCL12(unc-54/human Abeta peptide 1-42 minigene) + rol-6(su1006)]	https://cgc.umn.edu/strain/CL2006
CL2331	dvIs37 [myo-3p::GFP::A-Beta (3-42) + rol-6(su1006)]	https://cgc.umn.edu/strain/CL2331
BR5270	byIs161 [rab-3p::F3(delta)K280 + myo-2p::mCherry]	https://cgc.umn.edu/strain/BR5270
BR5271	yIs162 [rab-3p::F3(delta)K280 I277P I380P + myo-2p::mCherry]	https://cgc.umn.edu/strain/BR5271
DA2123	dIs2122 [lgg-1p::GFP::lgg-1 + rol-6(su1006)]	https://cgc.umn.edu/strain/DA2123

BC12921	Is10729 [rCes T12G3.1::GFP + pCeh361]	https://cgc.umn.edu/strain/BC12921

Table S2. Primer sequences for qRT-PCR.

**Gene name**	**Primer sequence（5'→3'）**
lgg-1 Forward	ACCCAGACCGTATTCCAGTG
lgg-1 Reverse	TCGTGATGGTCCTGGTAGAGT
bec-1 Forward	TCAGGACGAGCTTCATTCGC
bec-1 Reverse	GCTCCAACAGGATCTCTAATAACA
unc-51 Forward	CTATGTTGATCGCACAGACG
unc-51 Reverse	CTCCGTGCATTTGAGTAGGC
vps-34 Forward	TGTCGATTCTGCCTACCGGA
vps-34 Reverse	CGTTGTTCGATCTTGAAAATAGCC
cdc42 Forward	CTGCTGGACAGGAAGATTACG
cdc42 Reverse	CTCGGACATTCTCGAATGAAG
